# Factors influencing intention to obtain the HPV vaccine in South East Asian and Western Pacific regions: A systematic review and meta-analysis

**DOI:** 10.1038/s41598-018-21912-x

**Published:** 2018-02-26

**Authors:** Diviya Santhanes, Che Pui Yong, Yan Ye Yap, Pui San Saw, Nathorn Chaiyakunapruk, Tahir Mehmood Khan

**Affiliations:** 1 School of Pharmacy, Monash University Malaysia, Bandar Sunway, Selangor, Malaysia; 2 Center of Pharmaceutical Outcomes Research (CPOR), Department of Pharmacy Practice, Faculty of Pharmaceutical Sciences, Naresuan University, Phitsanulok, Thailand; 3 School of Pharmacy, University of Wisconsin, Madison, USA; 4 Asian Centre for Evidence Synthesis in Population, Implementation and Clinical Outcomes, (PICO), Health and Well-being Cluster, Global Asia in the 21st Century (GA21) Platform, Monash University Malaysia, Bandar Sunway, Selangor, Malaysia; 5 The Institute of Pharmaceutical Sciences (IPS), University of Veterinary & Animal Sciences (UVAS), Outfall road, Lahore, Pakistan

## Abstract

Since licensing in 2006, there has been poor uptake of the HPV vaccine among the targeted population in the South East Asia Region (SEAR) and Western Pacific Region (WPR). A systematic review was conducted to identify the studies exploring the relationship between factors and intention for HPV vaccination among women in SEAR and WPR countries. Nineteen studies were identified as suitable for qualitative synthesis, and three as suitable for meta-analysis. Most women had a positive intention to have an HPV vaccine (range 57%–85%). Having a positive intention to vaccinate was significantly higher among women not aware of HPV infection (OR: 1.34, 95% CI: 1.02–1.76) and HPV vaccine (OR: 1.57, 95% CI: 1.26–1.96). Lower knowledge level and less confidence in safety and efficacy of the vaccine, negatively affected intention to vaccinate. Perceiving the vaccine to be expensive, low perception of contracting HPV infection and cervical cancer, and lack of concrete recommendations from healthcare providers also negatively affected intention to vaccinate. This review suggests the decision-making processes of women in SEAR and WPR is influenced by the cost of vaccination, perceived efficacy and safety of vaccine, provision of information on vaccination, and the awareness about HPV infection and the HPV vaccine.

## Introduction

Human papillomavirus (HPV) has been recognised as the leading cause of cervical cancer, which in turn is the fourth-most prevalent cancer among women worldwide^1^. The World Health Organization’s (WHO) South East Asia Region (SEAR) and Western Pacific Region (WPR) have two of the highest burdens of cervical cancer^1–3^. In 2012, approximately 94,000 and 43,000 deaths from cervical cancer were recorded in the SEAR and the WPR, respectively^1^. HPV vaccines offer a promising breakthrough to curb the global burden of cervical cancer. Since 2006, the vaccine has been approved for use in over 100 countries. The United States was the first country to introduce a publicly funded HPV immunisation program^4^. In the WPR, Australia introduced its funded HPV immunisation schedule in 2007, followed by Malaysia in 2010 and Japan in 2011^4^. Conversely, there are no publicly funded HPV immunisation programs in the SEAR. An estimated 118 million women have been targeted for HPV immunisation worldwide and the number of fully vaccinated females in low- to upper-middle-income countries ranges from 1 million to 13.3 million, indicating a low uptake for HPV vaccination in these areas^4^. The lower response to HPV vaccination is reported to be due to vaccine hesitancy, which underpins reduced vaccine uptake and is defined as a delay in acceptance or refusal of vaccines despite availability of vaccine services^5^.

In this situation, it is vital to explore how the women in the SEAR and the WPR respond to the HPV vaccine; i.e., their intention to vaccinate. This systematic review is one of the first efforts to identify the factors influencing a woman’s intention to have the HPV vaccine. This review will also gauge the knowledge, beliefs and attitude towards the vaccine among women from the WPR and SEAR, as these are fundamental when making decisions about vaccination. To date, existing systematic reviews have summarised predictors of HPV vaccine intention and decision-making among women from the US, the UK, Australia and North America generally, and among African Americans in the US specifically^2,6,7^. However, none have demonstrated a clear association between influences and intention to vaccinate. Moreover, these systematic reviews have not explored the factors leading to vaccine hesitancy among women from the SEAR and WPR. These reviews also lack any quantitative analysis that associates the factors with intention to vaccinate for HPV^2,6,7^. This gap in the existing literature needs to be addressed, especially for the SEAR and WPR, which are regions in which a large female population of various ethnic, religious and socio-demographic backgrounds reside. The findings from this review will be valuable as the provision of statistical and qualitative data synthesis will further strengthen the association of factors that influence women’s decision-making regarding vaccination against HPV. Hence, this information will be of assistance in devising suitable interventions to improve access and acceptability to HPV vaccination in the SEAR and WPR.

## Results

### Study selection

**Figure 1:**
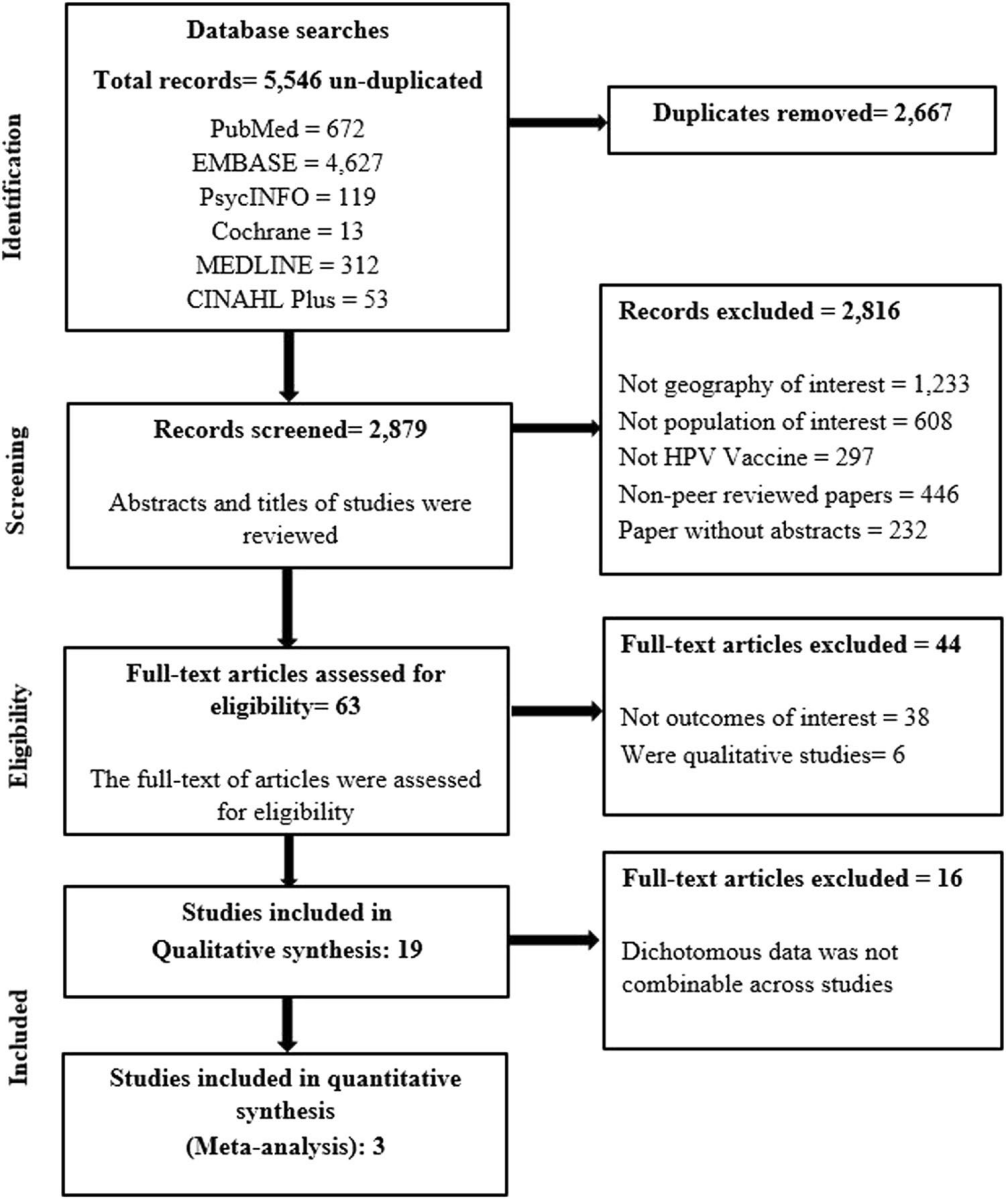
Flowchart for identification and inclusion of articles in systematic review.

Details of the study selection process are described in Fig. 1. A total of 5,546 research articles were identified through the initial search, of which 5,483 did not meet the inclusion criteria and were therefore excluded. Only 63 studies were considered eligible and examined in further detail. After further consideration, 44 articles were excluded: 6 were qualitative or mixed-methods synthesis studies, and 38 did not evaluate intention of receiving the HPV vaccine among their respondents. Studies investigating acceptability towards HPV vaccination or willingness to receive HPV vaccination were also included, as the assessment of HPV vaccine initiation was similar to studies investigating intention. A final number of 19 descriptive, cross-sectional studies met the inclusion criteria and were included for systematic review. Of these, 16 had marked diversity in measures of outcome reporting, and so were described in a qualitative synthesis manner. Three articles were identified as having similar comparator and outcome groups and underwent meta-analysis.

### Characteristics of included studies

The characteristics of all included studies are shown in Table 1. All 19 studies selected for review were descriptive and cross-sectional surveys in study design and used self-reported questionnaires to assess study outcomes. All studies were published in English and conducted post-licensure of the HPV vaccine (after 2006). The geographical distribution of these studies was widespread, with research carried out in China^8–10^, Hong Kong^11^, Japan^12,13^, Korea^14,15^, Taiwan^16,17^, Singapore^18^, Malaysia^19,20^, India^21^, Nepal^22^, Thailand^23–25^ and the Philippines^26^. Respondents were recruited from healthcare settings (including obstetrics and gynaecology clinics^21,23^, hospitals^12,19,21^ and health camps^22^, educational environments (including schools^10,11,25^, colleges^14,24^ and tertiary institutions^8,15,16,18,24^), random digit dialling^11^, community clusters^9,17,20,26^ and the internet^13^. The included studies all involved female respondents, with a total sample size of 22,533. Respondents consisted of mothers/parents with daughters eligible for HPV vaccination^10,12,13,19,22,25^, adult women^8,9,14–18,20,21,24,26^ and a combination^11,13^ (mothers and daughters). In most of the included studies, the primary outcomes assessed were factors such as awareness, knowledge and attitudes or health beliefs of respondents on practices and preventive measures of HPV infection^9,10,13,15–17,20–23,25^. Intention, acceptability or willingness to have the HPV vaccine were often secondary outcomes. Two studies investigated vaccine intention for the women themselves and their daughters^9,23^, seven studies for their daughters only^10–13,19,22,25^ and ten studies for getting vaccinating themselves^8,14–18,20,21,24,26^. Mothers were the decision-makers for daughters who were of adolescent age^9,12,13,19,23^. Studies among adult women in the SEAR and WPR demonstrated that they themselves made the decision about being vaccinated^8,14,17,18,24,26^. The influence of others (such as parents, partners/husbands) on a woman’s decision to have the HPV vaccine was not explored in most included studies in this review. Eleven studies reported that more than half of their respondents (Range: 56.5% – 84.6%) had a positive intention of undergoing the vaccination for themselves or their daughters^9,12,14–16,18–20,23–25^. In comparison, in seven studies fewer than half of the respondents (Range: 3.2% – 46%) had a positive intention to be vaccinated^8,10,11,13,17,21,26^. More women expressed an intention to have a vaccine (Range: 60% – 97.8%) if it was offered for free, as reported in four studies^17,19,22,25^.

**Table 1:** General characteristics of 19 included cross-sectional studies.

Study	Country	Objective	Respondents	Recruitment site	Sample size (response rate %)	Results obtained		NOS Score
						Primary outcome	Secondary outcome	
Charakorn C. *et al*.^23^	Thailand	Evaluation of knowledge about the Pap smear, HPV, and the HPV vaccine, and the acceptability of the vaccine to Thai women	Both mothers and their daughters	Hospital	536 (70%)	Poor knowledge of HPV infection and HPV vaccine	Positive intention for 77% and 84% of mothers and daughters, respectively	5
Choi HCW *et al*.^11^	Hong Kong	To provide a more representative and updated assessment on the acceptability of female adolescent HPV vaccination in Hong Kong	Schoolgirls aged between 11 and 18 years old, and mothers with daughter(s) ≤ 18 years old	Schoolgirls from 5 constituency areas and mothers through random-digit dialling telephone interviewing	Total of 1022 (39.3%) and 1005 (50.2%) mothers in 2008 and 2012 respectively. 2167 (96.2%) for schoolgirls’ survey	27.5% and 37.6% of mothers intended to vaccinate daughters’ in 2008 and 2012, respectively. 27.1% of schoolgirls in 2008.	Willingness to pay for full course of vaccination	6
Egawa-Takata T *et al*.^13^	Japan	To investigate why Japanese adolescent girls decline, continue or discontinue their HPV vaccination, how their mothers influence their decisions, and the mothers’ feelings about future HPV vaccination for their daughters	Mothers with daughters aged 10–18	Internet	2828 mothers (28.3%)	Mothers’ knowledge about HPV vaccine and attitude towards cervical cancer screening influenced their decision to get their daughters vaccinated	16% intended to get daughters vaccinated	5
Egawa-Takata T *et al*., 2016^12^	Japan	To investigate the prevalence of use of HPV vaccination in the daughters of obstetricians and gynecologists, and their attitudes related to the HPV vaccine and cervical cancer screening, to gain insights into their reasoning for or against recommending HPV vaccination for their daughters	Doctors’ daughters: HPV vaccinations before (2012) and after (2014) the adverse news releases	Hospital	264 (46%)	The number of vaccinated daughters was lower in 2014 than in 2012	64% intended to get daughters vaccinated	1
Gu C *et al*.^8^	China	To examine young women’s perceptions and acceptability of human papillomavirus vaccination and factors influencing acceptability in mainland China	Undergraduate female students year 1 to 4, aged 18 or above	Medical college	119 (94.3%)	44% of women intended to get vaccinated	Low awareness and knowledge of HPV vaccine and cervical cancer	7
Hsu YY *et al*.^16^	Taiwan	To examine health beliefs and intention to obtain HPV vaccination among undergraduate women in Taiwan	Full-time female undergraduate students attending 5 universities in Taiwan	Five universities in Taiwan	845	Poor awareness of HPV infection and HPV vaccine	63% of students intended to obtain HPV vaccine	7
Johnson DC *et al*.^22^	Nepal	Assessing knowledge and awareness of HPV, cervical cancer and HPV vaccines among rural and suburban women in Nepal	Women attending health camps	Khokana, a suburban community, and Sanphebagar, a rural community	749	Poor awareness and knowledge of HPV, cervical cancer and HPV vaccine	77.5% of women intended to obtain HPV vaccine if offered for free	6
Juntasopeepun P *et al*.^24^	Thailand	To examine knowledge and beliefs regarding HPV and cervical cancer and to predict vaccination intention among young women in Thailand	Thai women aged 18–24 years in Chiang Mai, Thailand	University	391	56.5% intended to receive the vaccine	Knowledge about HPV and cervical cancer were moderate	7
Kang HS *et al*.^14^	Korea	To examine the relationships between attitudes toward and intention to receive the HPV vaccination and intention to use condoms among Korean female college students	Female Korean college students	Sixteen colleges located in 16 regions across the nation	1600 (87%)	Intention to get vaccinated was not high, and women were not confident of the vaccine’s safety and changes in sexual behaviour	Intention gets lower when attitude is negative	5
Kang HY *et al*.^15^	Korea	To examine knowledge about HPV and attitudes towards HPV and HPV vaccination among Korean female undergraduate students	Female undergraduate students	University	339 (94.7%)	Awareness and knowledge of HPV was poor	Wanting more education about the vaccine, perceived severity and knowledge of HPV associated with intention	6
Kruiroongroj S *et al*.^25^	Thailand	To evaluate the willingness of Thai mothers to get their daughters vaccinated against HPV if it is free of charge (acceptance) or if it is not free of charge (willingness to pay), and to examine their current knowledge regarding HPV vaccine and cervical cancer	Female parents of adolescent girls aged 12–15	Secondary schools	1200 (71.7%)	Knowledge regarding the HPV vaccine was low	76.9% of parents intended to get vaccinated if offered for free and 68.9% were willing to pay if vaccine was not offered for free	3
Li J *et al*.^9^	China	To assess women’s knowledge about HPV and their acceptance of the vaccines	Women aged 14–59 living in metropolitan and rural regions of China	6 community clusters from 3 major cities and rural areas in 3 provinces	6024 (95.8)	Knowledge and awareness were low among women in both metropolitan and rural areas	84.6% of women intended to get vaccinated if the HPV vaccine was made available to them	7
Montgomery MP *et al*.^21^	India	To assess the knowledge, acceptability, attitudes and feasibility concerning HPV and cervical cancer among adult women in Dakshina Kannada district of the southern state of Karnataka, India	All women between the ages of 18 and 44	Family practice and obstetrics and gynaecology clinics and postnatal wards within the hospital Karnataka, India	225 (90%)	Knowledge and awareness related to HPV and cervical cancer was low	46% intended to receive the HPV vaccine	5
Sam IC *et al*.^19^	Malaysia	To determine the acceptability rates of HPV vaccination by Malaysian mothers for daughters	Mothers with at least one child under 18 years old attending outpatient clinics	University hospital	362	65.7% and 55.8% of mothers intended to get daughters and sons vaccinated, respectively	Knowledge of HPV and the HPV vaccine was low	5
Wong LP^20^	Malaysia	To assess the knowledge and attitudes towards HPV, HPV vaccination and cervical cancer among young women in rural settings	Women aged between 18 and 25 years and living in the household	Rural villages in the states of Perak and Pahang in Peninsular Malaysia	589 (84.7%)	Knowledge of HPV, HPV vaccination and cervical cancer were extremely poor	Two thirds of respondents professed an intention to receive the HPV vaccine	4
Yen CF *et al*.^17^	Taiwan	To explore awareness and acceptability of HPV vaccination and to identify factors influencing HPV acceptability among women with physical disabilities in Taiwan	Adult women aged 18–69 who were officially registered as having physical disabilities in Taipei City as of March 2009	As a part of a larger study on reported history of Pap smear tests, health experiences, perceptions of cervical cancer and HPV vaccination among women with physical disabilities in Taiwan.	438	Awareness of HPV vaccine was poor	Only 3.2% intended to obtain the vaccine. Intention increased up to 60% if the vaccine was offered for free	4
Young AM *et al*.^26^	Philippines	To examine attitudes toward and acceptability of HPV vaccination among a community-based sample of women in the Philippines	Women aged from 18 to 52 years old	Three communities in the Central Visayan region	435	54% intended to receive HPV vaccine at low price, whereas only 30% and 31% intended to receive at moderate and high prices, respectively	Mothers and partners were influential in vaccination decisions together with access to transportation, social support and benefits of vaccination	4
Yu Y *et al*.^10^	China	To investigate awareness and knowledge of HPV/the HPV vaccine and potential acceptance of the HPV vaccine among mothers with a teenage daughter in Weihai, Shandong, China	Mothers of daughters aged 9–17 years old	Weihai, China	1578 (85.3%)	Awareness and knowledge of HPV/HPV vaccine was poor	Only 26.49% of mothers intended to get their daughters vaccinated	5
Zhuang QY *et al*.^18^	Singapore	To describe the knowledge, attitudes and practices of young women regarding HPV vaccination	Female students attending a tertiary institution in Singapore	University	255	Among the unvaccinated participants (n = 230), 41.7% had no intention to receive the vaccine and 27.0% cited lack of information as a major barrier to HPV vaccination	Knowledge of HPV and cervical cancer was found to be low	6

#### Quality assessment of included studies

The quality assessment of all included studies in the review is described in Supplementary Table S3. Eight included studies were of good quality^8,9,11,15,16,18,22,24^, with scores ranging from 6 to 7; six studies had average quality^10,13,14,19,21,23^ with a score of 5; and the remaining five studies were of poor quality, with scores ranging from 3 to 4^12,20,25,26^.

### Factors influencing intention to obtain HPV vaccine

#### Knowledge on preventive measures of HPV infection and its complications

Having awareness of HPV, the HPV vaccine, cervical cancer or genital warts does not necessarily mean good knowledge of these. In addition, the type and number of questionnaire items used to assess the knowledge level of women differed markedly between studies, so it was challenging to combine the results. Overall, five studies reported that women having better knowledge on preventive measures of HPV infection and its complications are more likely to express intention to receive the vaccine^8,10,15,20,23^. Only one study, by Juntasopeepun, *et al*.^24^, reported that there was no significant association between knowledge level of HPV and cervical cancer and intention to get vaccinated, among young Thai women^24^.

#### Perceived susceptibility to HPV and cervical cancer

Eight studies associated women’s perception of contracting HPV and cervical cancer in the future to intention to vaccinate^8,9,13,15,16,21,24,26^. Women who perceive themselves to be at risk of contracting HPV infection and cervical cancer were more likely to have the vaccine than women who do not. In addition, not many women felt that they are at risk of getting HPV or cervical cancer. Three studies reported, respectively, that only 4% (n = 8) of Indian women, 9.5% (n = 37) of Philippine women and 20% of Malaysian women felt that they were at risk of getting HPV^20,21,26^.

#### Perceived seriousness of disease

Three studies measured the perceptions of respondents to disease severity of HPV infection and cervical cancer^15,16,24^. Two studies stated that female university students who perceived cervical cancer as very serious had higher chances of getting vaccinated against HPV compared to those who thought otherwise^15,16^. The study by Juntasopeepun, *et al*.^24^, among college-aged Thai women did not find a significant association between women’s perception of severity of cervical cancer and intention to vaccinate.

#### Concerns about side effects of HPV vaccine

A total of seven studies associated concerns about side effects of the HPV vaccine with non-receipt of HPV vaccination. Six of these studies found that a female parent or a young women’s concern about the adverse effects of the vaccine negatively affected vaccination intentions^8,10,12,20,25^. Only one study by Hsu, *et al*.^16^, reported that the HPV vaccine’s adverse effects had no significant association with intention to vaccinate.

#### Confidence in the efficacy of the vaccine

Four studies predicted that women’s confidence in the efficacy of the HPV vaccine would affect the intention to vaccinate. Women’s intention to receive the HPV vaccine increases if they are confident in the efficacy of the vaccine in combating cervical cancer, as demonstrated by two studies^8,13^. Mothers who doubted the efficacy of the vaccine were not likely to accept the vaccination for their daughters^10,19^.

#### Cost of HPV vaccine

Five studies associated cost concerns with intention to vaccinate. Of these, three studies reported that the cost of the vaccine was a barrier to vaccination^16,19,24^. One study in India stated that only 12% (n = 202) of women would agree to pay for all three doses of HPV vaccine, at a cost of $360 US dollars^21^. On the contrary, the cost of the vaccine was not a profound barrier among mothers in Shandong, China: only 3.79% (n = 1,160) of them thought the vaccine was too expensive^10^.

#### Concerns about risky sexual behaviour

Concerns about risky sexual behaviour were only shown among a minority of female Thai and Korean college students when asked for their reasons for non-vaccination against HPV^14,25^.

#### Recommendation from others

Women who received a recommendation from others to opt for vaccination were more likely to accept the vaccine compared to those who did not receive any recommendation. The healthcare provider was cited as the most trusted source of recommendation^16,20,24,26^. Women who received recommendations from a healthcare provider were more likely to undergo HPV vaccination^16,26^.

#### Educational need

Needing more information on HPV-related aspects was shown to have a profound effect in two studies^15,18^. Lack of information was the main reason why Singaporean and Korean university students refused vaccination. Only two studies reported concerns about the source of the vaccine^9,10^. Doubts about the sources of the vaccine were the major reason for unwillingness to get vaccinated among Chinese women in both rural and metropolitan areas and a minority of mothers in Shandong, China^10^.

#### Awareness of HPV infection and HPV vaccine

**Figure 2:**
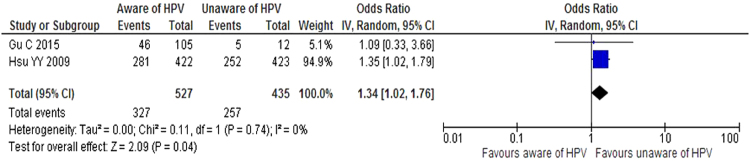
Forest plot of the two studies estimating the effect of awareness of HPV infection on positive intention to have the HPV vaccine.

Of the 19 included studies, only two examined the relationship between awareness of HPV infection and positive intention to vaccinate^8,16^. Women who are unaware of HPV have a higher chance 1.34 times greater [1.34 (CI 95% 1.02—1.76)] than those aware of HPV of getting vaccinated (Fig. 2), with no heterogeneity identified within and between the included studies [Tau^2^ = 0.00, Chi = 0.11, df = 1; I^2^ = 0%].

**Figure 3:**
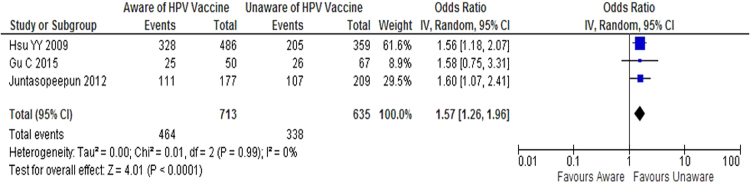
Forest plot of the three studies estimating the effect of awareness of HPV vaccine on positive intention to have the HPV vaccine.

Three studies examined the relationship between awareness of HPV vaccine and intention to vaccinate^8,16,24^. The likelihood of positive intention to undergo HPV vaccination is 1.57 times [1.57 (CI 95% 1.26—1.96)] higher among women unaware of the HPV vaccine compared to women aware of HPV vaccine (Fig. 3). Again, no heterogeneity was found between these three studies [Tau^2^ = 0.00, Chi = 0.01, df = 2; I^2^ = 0%].

## Discussion

This is the first systematic review that scrutinizes the literature on factors influencing the intention of women in countries in the SEAR and WPR to get HPV vaccines. Investigating their intention to receive HPV vaccines and how it is affected by influential factors is a useful insight to devise strategies that can remove the barriers involved and facilitate a more positive intention of vaccinating against HPV.

In regions of high cervical cancer mortality such as SEAR and WPR, this review revealed that young women and female parents generally have positive intentions (57% to 65% of respondents) to get themselves or their children vaccinated against HPV. Despite this, concerns and barriers were identified when a women hesitates to obtain HPV vaccine.

Based on the results from our meta-analysis, women unaware of HPV infection and the availability of HPV vaccine are respectively 1.34 and 1.57 times more likely to receive vaccination. It is possible that women in the ‘unaware’ group possessed limited information, for example from media sources or friends and may consider the HPV as a bigger health concern in comparison to those who are more aware of the subject. The mixed views on HPV vaccination makes it difficult for parents and young women to make informed decisions^27^. Our findings of a lower intention to vaccinate associated with improved awareness are consistent with those of Trim, *et al*.^28^, obtained from a systematic review of mostly American and European studies (81.1% of included studies). Although the percentage of parents who have heard about HPV rose over time from 60% (2005) to 93% (2009), the intention to vaccinate declined from 80% (2008) to 41% (2011)^28^. The reason for this decline was that parents were concerned about the safety of the vaccine and wanted more information^28^. This further affirms that the decision-making process for the HPV vaccination is multifactorial, and an assessment of what hinders women from taking the HPV vaccine needs to consider all the influential factors^29^. Thus, it is essential to understand that being aware of HPV vaccination and the decision to be vaccinated against the HPV are two different issues, and ‘being well aware’ does not necessarily indicate that an individual will opt for vaccination for their children. Yang, *et al*.^30^ reported that personal beliefs and understanding about vaccination are the main factors that dictate the decision not to vaccinate or to seek exemption from mandatory vaccination among well-educated respondents in California. Perlman, *et al*.^29^ identified high levels of acceptability of the HPV vaccine despite low levels of knowledge and mixed levels of awareness of cervical cancer, HPV and the HPV vaccine^29^. It is hard to judge why lower awareness led to higher acceptability of the HPV vaccine, however, a possibility is that due to lack of knowledge individuals may consider the HPV as a bigger health concern in comparison to those who are well educated or more aware of the HPV and its vaccine. Although the fall in HPV vaccine uptake with awareness may be considered counterintuitive, it affirms the multifactorial nature of the decision-making process and underlines the need for assessment of all influential factors. It may be wise to focus on ‘belief modification’ of the public to improve vaccine uptake by targeting information on the importance and safety of vaccines to specific sections of the community^30^.

From our study, it was evident that wanting more information on HPV vaccination was a reason for refusal among women in the SEAR and WPR. Reviews by Holman, *et al*.^6^, and Kessels, *et al*.^7^, in high-income countries (the US, the UK, Canada and Australia) associated parental satisfaction with the amount and quality of information with vaccine uptake, whereas vaccine refusal was linked to dissatisfaction with information^6,7^.

Safety concerns about the vaccine were also found to be profound. Most included studies revealed that women concerned about the side effects of the vaccine had a more negative intention to undergo vaccination^8,10,12,20,25^. This resonates with findings from Hopkins, *et al*.^31^, and Kessels, *et al*.^7^, here parental concerns about vaccine safety and side effects negatively affected vaccine initiation.

Our review revealed that women in the SEAR and WPR had doubts about the efficacy of the HPV vaccine. A lack of confidence in the vaccine’s ability to prevent cervical cancer was associated with a more negative intention to get vaccinated^8,10,13,19^. This negative belief was also reflected in other countries; for example, the US and in Africa, where increased confidence in the vaccine led to a positive intention to have the HPV vaccine^32–35^. Effectiveness of other vaccines such as polio and measles influenced respondents’ receipt of the HPV vaccine, as reported by both Katahoire, *et al*.^36^ and Ports, *et al*.^37^. In addition, parents from developed countries who had previously had their child vaccinated against meningitis or had a positive belief in the efficacy of the vaccine were more likely to get their child vaccinated against HPV^28^. Confidence in the efficacy of the HPV vaccine was cited in only four studies in this review. In these studies, women who perceived the vaccine to be effective in combating cervical cancer and HPV infection were more likely to receive the vaccination. This was only discussed in African studies, but the results were very varied making it difficult to come to a conclusion^38^.

The cost of the vaccine was a significant barrier for women in the SEAR and WPR, as the HPV vaccine is not funded in most countries in these regions^39,40^. To date, the only countries that have introduced the HPV vaccine in their national immunisation schedule since its licensure are Australia, Bhutan, Brunei Darussalam, Japan, Malaysia, Singapore, Micronesia and Palau, which together comprise 21% (8/38) of countries in the two regions^41–43^. As both the SEAR and WPR mostly consist of low- and middle-income countries (LMIC), setting up a publicly funded HPV immunisation program can be costly, and access to cervical cancer screening programs and healthcare facilities can be a significant issue^31,44^. In a study by Ferrer, *et al*.^39^, financial concerns came up in high-income countries (HIC) such as the US, Hong Kong and Sweden^39^. Women in the US were reluctant to go for an HPV vaccination due to the lack of reimbursement by insurance companies, and women in Hong Kong (one of the countries that does not offer free HPV vaccination) thought the vaccine was too expensive^33,39^. The HPV vaccine is available to girls aged 13 to 17 in Sweden on a paying basis, which is a barrier for poor families^39^. A study by Cunningham, *et al*.^38^, in Africa on the intention to get the HPV vaccine also revealed cost concerns as an important barrier. Some participants thought the vaccine should be offered for free, while others indicated willingness to pay, but substantially less than the actual cost^38^. However, cost was not an evident barrier where the vaccine was offered for free, such as in Australia, where the vaccine was provided free for a limited time, prompting young women to get vaccinated^39,40^.

In both the SEAR and WPR, women who perceived they are at low risk of contracting HPV cervical cancer were less likely to get vaccinated compared to women who thought otherwise. Many women in both these regions also did not feel they are at risk of acquiring HPV infection and cervical cancer, although the reason for this was not explored in all relevant studies. The minority of women who felt they were at risk was reported in the international literature as well. Low perception of the risk of HPV acquisition and misperceptions of need based on sexual activity was also reported in the review by Holman, *et al*.^6^, on HIC. In addition, women who perceived themselves at risk of getting HPV infection or cervical cancer were more likely to have the vaccine. In comparison, studies in Africa reported otherwise: most parents and adults perceived the risk of HPV infection of cervical cancer for their daughters as high, ranging from 41% to 78%, but its relationship with intention to vaccinate was mixed^38^. Women in the SEAR and WPR who saw cervical cancer as a serious disease had higher chances of getting vaccinated. This finding resonated with Trim, *et al*.^28^, and Cunningham, *et al*.^35^, which were literature reviews of HIC and Africa respectively, and which showed that those who recognized cervical cancer as a disease of higher severity were also more likely to accept the vaccine.

Concerns about initiating early or risky sexual behaviour among their daughters did not prominently appear in our review, with only two studies citing this concern. In comparison with other regions, this concern appeared mostly among Asian mothers residing in developed countries^39^. Irrespective of geography and delivery setting of the HPV vaccine, cultural sensitivity was profound among women when deciding to allow their daughters to undergo vaccination for cervical cancer. Muslim and Asian mothers in the UK and Australia were concerned that giving permission their daughters to be vaccinated would be seen as a sign of approval for sex before marriage^45,46^. Some parents dismissed this viewpoint, such as Alaskan and Australian parents, when allowing their child to be vaccinated against HPV^39,46,47^.

Support from family and friends and a physician’s recommendation are also important when women in the SEAR and WPR decide about having the HPV vaccine. Regardless of a country’s income status and cervical cancer mortality rate, women in the SEAR and WPR had the same concerns and barriers as women from other countries regarding intention to have the HPV vaccine. A women receiving support from her parents, encouragement from friends or recommendation from a physician is more likely to have the vaccine. Receiving a physician’s recommendation or discussing the HPV vaccine with a physician was associated with vaccine acceptance and initiation in numerous studies from HIC as reported by Holman, *et al*.^6^. However, it was also reported in the same study that parents frequently cited not having a physician’s recommendation as a reason for not getting their child vaccinated. Studies in Africa also stated similar findings: a doctor’s recommendation was positively associated with intention to vaccinate^38^. There is a need for a more tailored health-promotion program for HPV vaccination. In this, it would be useful that parents and young women understand why HPV vaccination is recommended at a young age, the benefits of taking the HPV vaccine and how it outweighs the risks involved (such as adverse effects), the risk factors involved in acquiring HPV infection, and the efficacy of the HPV vaccine in preventing genital warts and cervical cancer. Abolishing the cultural and religious barriers may be impossible, but pitching the vaccine as a cervical cancer vaccine rather than the HPV vaccine may help improve uptake. In addition, it is recommended that parents and young women be educated on why the HPV vaccine needs to be obtained at a young age rather than later. Although there were uncertainties about the benefits of vaccinating women aged 15 to 45 years^48^, Gardasil was reported to remain highly effective in preventing HPV-related diseases in females of this age group who were HPV-naïve^49^ but less effective in those who may have had a previous HPV infection^50^. Thus, GPs should still consider older women who may benefit from HPV vaccination by taking into account of their previous exposure and the risk of future exposure to HPV. Reducing the cost of the vaccine also needs to be considered; support from donations and government subsidies would be useful to help reduce the cost of getting the HPV vaccine. Access to healthcare facilities among populations in rural settings needs to be investigated, and suitable interventions are needed to ensure the populations there are also educated about the HPV vaccine.

### Strengths and limitations of this review

This systematic review provides a comprehensive summary of the concerns and barriers that women in the SEAR and WPR have when making informed decisions on HPV vaccination. It is a good reflection of factors contributing to HPV vaccine hesitancy in populations that are at high risk of developing cervical cancer and HPV infection despite the availability of vaccine services. However, this review is limited by studies that mostly recruited populations from cities or urban areas, so it cannot be generalised to other populations such as rural residents, ethnic minorities or immigrants. Further, this review may not be applicable to all LMICs, as there may be pilot schemes or GAVI projects in which vaccine hesitancy cannot be applied.

## Conclusion

Intention to vaccinate was generally positive among women from the SEAR and WPR. The main factors that led to a negative intention to receive the HPV vaccine were the cost of vaccination, concerns about efficacy and safety of the vaccine, lack of information on vaccination, and lack of awareness about HPV infection and the HPV vaccine. Future research should involve investigations of the actual uptake and completion of HPV vaccination, and barriers to receiving the HPV vaccine (rural areas, lower socioeconomic status, and ethnic minorities). Health promotion programs for the HPV vaccine should clarify women’s risks of acquiring HPV infection and the benefits of getting vaccinated, while being culturally sensitive.

## Methods

### Research protocol and registration

A systematic review was performed using Preferred Reporting Items for Systematic Reviews and Meta-Analyses (PRISMA). The protocol for this systematic review is registered under Centre for Reviews and Dissemination (PROSPERO) and is available from: http://www.crd.york.ac.uk/PROSPERO/display_record.asp?ID=CRD42016035749^51^.

### Search strategy

All the relevant studies from inception till 31^st^ December 2016 were considered for inclusion. Six databases, namely Pubmed, EMBASE (Ovid), PsycINFO (Ovid), Cochrane, MEDLINE (EBSCOhost) and CINAHL Plus (EBSCOhost), were systematically searched using search terms and keywords by three independent reviewers (DS, CP, YY). To capture all relevant influential factors involved in women’s decision-making processes for having the HPV vaccine, the following Mesh terms were used in Pubmed, connected with Boolean phrase AND: “papillomavirus infections”, “female”, “papillomavirus vaccines”, “health knowledge, attitudes, practice”. Details of search strategies in the remaining databases are provided in the Supplementary Table S1. No language restrictions were imposed, but geographical restriction was applied in some databases.

### Inclusion criteria

Upon completion of the search across six databases, the following inclusion criteria were used to identify the potential papers for qualitative and qualitative synthesis: (1) A quantitative study that examined the factors influencing women’s decision-making processes in obtaining the HPV vaccine and their intention to obtain the HPV vaccine; (2) The study was conducted in WHO-defined SEAR and WPR; and (3) The study recruited participants eligible for HPV immunisation; i.e., girls aged 9–13 years old and girls aged ≥ 15 years old. Parents who had daughter’s eligible for HPV immunisation were also included.

### Study Selection

Studies meeting the inclusion criteria were further screened for duplication by three independent reviewers (DS, YYY and CPW). After reaching a final consensus about the article numbers, the full-text articles of the studies were then screened for the final inclusion. Disagreements were resolved by discussion and consensus between three reviewers (DS, YYY and CPW). If no agreement could be reached, the remaining two reviewers (TK and PSS) made a decision. The identification and inclusion of articles is depicted in a PRISMA flow diagram (Fig. 1).

### Data Extraction Process

The data extraction form was developed in an Excel spreadsheet. The form was piloted from two trial reports to ensure suitability for use. A completed extraction form is attached in Supplementary Table 2. Three reviewers (DS, YYY and CPW) extracted the relevant data from each included study, and two review authors (TK and PSS) checked the extracted data. Disagreements were resolved by discussion and consensus among all authors. No attempt was made to seek additional information from the included primary studies.

### Outcome of interest

The primary outcome of interest for this systematic review was the intention to vaccinate for HPV. Any other relevant information that encompassed awareness and knowledge of HPV-related aspects, factors accepting or rejecting vaccination, sources of information, and attitudes and beliefs, were also considered for further comparison between the studies.

### Quality assessment of included studies

All the studies included in this systematic review were observational. The quality of the included observational studies was assessed using the Newcastle–Ottawa scale (NOS) which reports the quality of studies on a scale of 0 to 10^52^. One author (DS) evaluated the quality of included studies on both study and outcome levels (Supplementary Table S3). This was subsequently verified and crossed-checked by TMK and PS.

### Synthesis of results (quantitative)

Articles presenting data relevant to the outcomes of interest were further subjected to Meta-analysis using Review Manager (RevMan), version 5.3. A random effects model was applied during analysis because the true effect size varies according to differences in population from study to study. The odds ratio (OR) was computed together with inverse variance and 95% confidence interval (CI) for calculation. We tested for heterogeneity to measure inconsistency of effects across studies. The level of heterogeneity between and within studies was determined as either low (25%), moderate (50%) or high (75%)^53^. Studies with binary data that was not combinable due to marked variation between studies were synthesised narratively.

## Electronic supplementary material

Supplementary Table S1

Supplementary Table S2

Supplementary Table S3

## References

[1] GLOBOCAN 2012: Estimated Cancer Incidence, M. A. P. W. *Cervical Cancer*, *Estimated Incidence*, *Mortality and Prevalence Worldwide in* 2012, http://globocan.iarc.fr/Pages/fact_sheets_cancer.aspx (2012).

[2] Hendry M, Lewis R, Clements A, Damery S, Wilkinson C (2013). “HPV? Never heard of it!”: a systematic review of girls’ and parents’ information needs, views and preferences about human papillomavirus vaccination. Vaccine.

[3] World Health Organization. Sexually transmitted infection (STI) Fact Sheet, http://www.who.int/mediacentre/factsheets/fs110/en/ (2016).

[4] Bruni, L. *et al*. Global estimates of human papillomavirus vaccination coverage by region and income level: a pooled analysis. *The Lancet Global Health* **4**, e453–e463, 10.1016/S2214-109X(16)30099-7. 10.1016/S2214-109X(16)30099-7 27340003

[5] World Health Organization. Report of the Sage Working Group on Vaccine Hesitancy, http://www.who.int/immunization/sage/meetings/2014/october/1_Report_WORKING_GROUP_vaccine_hesitancy_final.pdf (2014).

[6] Holman DM (2014). Barriers to human papillomavirus vaccination among us adolescents: A systematic review of the literature. JAMA Pediatrics.

[7] Kessels SJM (2012). Factors associated with HPV vaccine uptake in teenage girls: A systematic review. Vaccine.

[8] Gu C, Niccolai LM, Yang S, Wang X, Tao L (2015). Human papillomavirus vaccine acceptability among female undergraduate students in China: the role of knowledge and psychosocial factors. Journal of Clinical Nursing.

[9] Li J (2009). Knowledge and attitudes about human papillomavirus (HPV) and HPV vaccines among women living in metropolitan and rural regions of China.(Report). Vaccine.

[10] Yu Y (2016). Human Papillomavirus Infection and Vaccination: Awareness and Knowledge of HPV and Acceptability of HPV Vaccine among Mothers of Teenage Daughters in Weihai, Shandong, China. PloS one.

[11] Choi, C., Woo, P., Jit, M., Leung, G. & Wu, J. Acceptability and uptake of female adolescent HPV vaccination in Hong Kong: a survey of mothers and adolescents. **32**, 78–84 (2013). 10.1016/j.vaccine.2013.10.068 24188759

[12] Egawa-Takata T (2016). Human papillomavirus vaccination of the daughters of obstetricians and gynecologists in Japan. International journal of clinical oncology.

[13] Egawa-Takata T (2015). Survey of Japanese mothers of daughters eligible for human papillomavirus vaccination on attitudes about media reports of adverse events and the suspension of governmental recommendation for vaccination. Journal of Obstetrics and Gynaecology Research.

[14] Kang HS, Moneyham L (2010). Attitudes toward and intention to receive the human papilloma virus (HPV) vaccination and intention to use condoms among female Korean college students. Vaccine.

[15] Kang H-Y, Kim J-S (2011). Knowledge, Attitudes of Human Papillomavirus Vaccine, and Intention to Obtain Vaccine Among Korean Female Undergraduate Students. Women & Health.

[16] Hsu Y-Y (2009). Intention to obtain human papillomavirus vaccination among Taiwanese undergraduate women. Sexually Transmitted Diseases.

[17] Yen C-F (2011). The Acceptability of Human Papillomavirus (HPV) Vaccination among Women with Physical Disabilities. Research in Developmental Disabilities: A Multidisciplinary Journal.

[18] Zhuang QY, Wong RX, Chen WM, Guo XX (2016). Knowledge, attitudes and practices regarding human papillomavirus vaccination among young women attending a tertiary institution in Singapore. Singapore medical journal.

[19] Sam, I. C. *et al*. Maternal Acceptance of Human Papillomavirus Vaccine in Malaysia. *Journal of Adolescent Health* **44**, 610–612, 10.1016/j.jadohealth.2008.11.014. 10.1016/j.jadohealth.2008.11.014 19465327

[20] Wong L (2011). Knowledge and Attitudes About HPVInfection, HPV Vaccination, and Cervical Cancer Among Rural Southeast Asian Women. Official Journal of the International Society of Behavioral Medicine.

[21] Montgomery M, Dune T, Shetty P, Shetty A (2015). Knowledge and Acceptability of Human Papillomavirus Vaccination and Cervical Cancer Screening among Women in Karnataka, India. Journal of Cancer Education.

[22] Johnson DC (2014). Knowledge and awareness of human papillomavirus (HPV), cervical cancer and HPV vaccine among women in two distinct Nepali communities. Asian Pacific journal of cancer prevention: APJCP.

[23] Charakorn C, Lertkhachonsuk RS, Thanapprapasr A, Chittithaworn D, Wilailak S (2011). S. Knowledge of Pap smear, HPV and the HPV vaccine and the acceptability of the HPV vaccine by Thai women.(Report). Asia-Pacific Journal of Clinical Oncology.

[24] Juntasopeepun P, Davidson PM, Suwan N, Phianmongkhol Y, Srisomboon J (2011). Human papillomavirus vaccination intention among young women in Thailand. Asian Pacific journal of cancer prevention: APJCP.

[25] Kruiroongroj S, Chaikledkaew U, Thavorncharoensap M (2014). Knowledge, acceptance, and willingness to pay for human papilloma virus (HPV) vaccination among female parents in Thailand. Asian Pacific journal of cancer prevention: APJCP.

[26] Young AM (2010). HPV vaccine acceptability among women in the Philippines. Asian Pacific journal of cancer prevention: APJCP.

[27] McKee C, Bohannon K (2016). Exploring the Reasons Behind Parental Refusal of Vaccines. The Journal of Pediatric Pharmacology and Therapeutics: JPPT.

[28] Trim K, Nagji N, Elit L, Roy K (2012). Parental Knowledge, Attitudes, and Behaviours towards Human Papillomavirus Vaccination for Their Children: A Systematic Review from 2001 to 2011. Obstetrics and Gynecology International.

[29] Perlman S (2014). Knowledge and Awareness of HPV Vaccine and Acceptability to Vaccinate in Sub-Saharan Africa: A Systematic Review. PLoS ONE.

[30] Yang YT, Delamater PL, Leslie TF, Mello MM (2016). Sociodemographic Predictors of Vaccination Exemptions on the Basis of Personal Belief in California. Am J Public Health.

[31] Hopkins TG, Wood N (2013). Female human papillomavirus (HPV) vaccination: Global uptake and the impact of attitudes. Vaccine.

[32] Cui Y, Baldwin SB, Wiley DJ, Fielding JE (2010). Human papillomavirus vaccine among adult women: disparities in awareness and acceptance. American journal of preventive medicine.

[33] Zimet GD (2000). Acceptability of Human Papillomavirus Immunization. Journal of Women’s Health & Gender-Based Medicine.

[34] Brewer NT, Fazekas KI (2007). Predictors of HPV vaccine acceptability: A theory-informed, systematic review. Preventive Medicine.

[35] Cunningham MS, Davison C, Aronson KJ (2014). HPV vaccine acceptability in Africa: a systematic review. Prev Med.

[36] Katahoire RA (2008). An Assessment of the Readiness for Introduction of the HPV Vaccine in Uganda. African Journal of Reproductive Health/La Revue Africaine de la Santé Reproductive.

[37] Ports KA, Reddy DM, Rameshbabu A (2013). Barriers and Facilitators to HPV Vaccination: Perspectives from Malawian Women. Women & Health.

[38] Cunningham MS, Davison C, Aronson KJ (2014). HPV vaccine acceptability in Africa: A systematic review. Preventive Medicine.

[39] Ferrer HB, Trotter C, Hickman M (2014). & Audrey, S. Barriers and facilitators to HPV vaccination of young women in high-income countries: a qualitative systematic review and evidence synthesis. BMC Public Health.

[40] Brotherton JML, Piers LS, Vaughan L (2015). Estimating human papillomavirus vaccination coverage among young women in Victoria and reasons for non-vaccination. Sexual Health.

[41] World Health Organization. *Countries in WHO South-East Asia Region*, http://www.who.int/about/regions/searo/en/ (2017).

[42] World Health Organization. Immunization, Vaccines and Biologicals, http://www.who.int/immunization/diseases/hpv/decision_implementation/en/ (2013).

[43] World Health Organization. *Countries in the WHO Western Pacific Region* http://www.who.int/about/regions/wpro/en/ (2017).

[44] Kane, M. A., Sherris, J., Coursaget, P., Aguado, T. & Cutts, F. Chapter 15: HPV vaccine use in the developing world. *Vaccine* 24, Supplement **3**, S132–S139, 10.1016/j.vaccine.2006.05.128 (2006). 10.1016/j.vaccine.2006.05.128 16950000

[45] Marlow LAV, Wardle J, Waller J (2009). Attitudes to HPV vaccination among ethnic minority mothers in the UK: An exploratory qualitative study. Human Vaccines.

[46] Robbins SC, Bernard D, McCaffery K, Brotherton JM, Skinner SR (2010). “I just signed”: Factors influencing decision-making for school-based HPV vaccination of adolescent girls. Health psychology: official journal of the Division of Health Psychology, American Psychological Association.

[47] Toffolon-Weiss M (2008). Alaska Native parental attitudes on cervical cancer, HPV and the HPV vaccine. International journal of circumpolar health.

[48] Mazza D, Petrovic K, Grech C, Harris N (2014). HPV vaccination in women aged 27 to 45 years: what do general practitioners think?. BMC Women’s Health.

[49] Castellsague X (2011). End-of-study safety, immunogenicity, and efficacy of quadrivalent HPV (types 6, 11, 16, 18) recombinant vaccine in adult women 24-45 years of age. Br J Cancer.

[50] Leval A (2013). Quadrivalent human papillomavirus vaccine effectiveness: a Swedish national cohort study. J Natl Cancer Inst.

[51] T M. Khan, C. P. Wong. & Yap, Y. Y. *Vaccine hesitancy towards Human Papillomavirus (HPV) vaccination in South East Asia Region (SEAR) and Western Pacific Region (WPR): a systematic review* http://www.crd.york.ac.uk/PROSPERO/display_record.asp?ID=CRD42016035749. 10.1080/21645515.2017.1381811 PMC5791566 28933635

[52] Wells, G. A. *et al*. The Newcastle-Ottawa Scale (NOS) for assessing the quality of nonrandomised studies in meta-analyses, http://www.ohri.ca/programs/clinical_epidemiology/oxford.asp.

[53] Borenstein, M., Hedges, L. V., Higgins, J. P. T. & Rothstein, H. R. Rothstein. *Introduction to Meta-Analysis.* 107–125 (Wiley Publications, 2009).

