# Supplementary material for: Factors influencing intention to obtain the HPV vaccine in South East Asian and Western Pacific regions: A systematic review and meta-analysis
Source: Sci Rep. 2018 Feb 26;8:3640. doi: 10.1038/s41598-018-21912-x (PMC5832144; doi:10.1038/s41598-018-21912-x)
Supplement: Supplementary file 1 — Supplementary Table S1 [file 41598_2018_21912_MOESM1_ESM.pdf]

## Appendix 1: Search strategy

| | |
|---|---|
| **PubMed**<br><br>Total results=672 | (("papillomavirus infections"[MeSH Terms] AND "female"[MeSH Terms]) AND "papillomavirus vaccines"[MeSH Terms]) AND "health knowledge, attitudes, practice"[MeSH Terms] |
| **Embase**<br><br>Total results=4, 627 | (human papillomavirus and vaccination and female).mp. [mp=title, abstract, heading word, drug trade name, original title, device manufacturer, drug manufacturer, device trade name, keyword, floating subheading] |
| **PsycINFO**<br><br>Total results=119 | (human papillomavirus and vaccination and female).mp. [mp=title, abstract, heading word, table of contents, key concepts, original title, tests & measures] |
| **Cochrane**<br><br>Total results=13 | "human papilloma virus":ti,ab,kw and "female":ti,ab,kw and "vaccination":ti,ab,kw (Word variations have been searched) |
| **MEDLINE [EBSCO HOST]**<br><br>Total results=312 | S1: TX human papillomavirus AND TX female AND TX vaccination<br>S2: TX human papillomavirus AND TX female AND TX vaccination<br>**Narrow by SubjectGeographic:** - republic of korea<br>**Narrow by SubjectGeographic:** - asia<br>**Narrow by SubjectGeographic:** - malaysia<br>**Narrow by SubjectGeographic:** - taiwan<br>**Narrow by SubjectGeographic:** - thailand<br>**Narrow by SubjectGeographic:** - hong kong<br>**Narrow by SubjectGeographic:** - vietnam<br>**Narrow by SubjectGeographic:** - japan<br>**Narrow by SubjectGeographic:** - india<br>**Narrow by SubjectGeographic:** - china<br>**Narrow by SubjectGeographic:** - australia<br>**Search modes** - Boolean/Phrase |
| **CINAHL Plus**<br><br>Total results=53 | human papillomavirus AND vaccination AND female<br><br>Narrow by SubjectGeographic: - asia<br>Narrow by SubjectGeographic: - australia & new zealand<br>Search modes - Boolean/Phrase |
