# Supplementary material for: Factors influencing intention to obtain the HPV vaccine in South East Asian and Western Pacific regions: A systematic review and meta-analysis
Source: Sci Rep. 2018 Feb 26;8:3640. doi: 10.1038/s41598-018-21912-x (PMC5832144; doi:10.1038/s41598-018-21912-x)
Supplement: Supplementary file 3 — Supplementary Table S3 [file 41598_2018_21912_MOESM3_ESM.pdf]

Supplementary table S3: Quality assessment of included studies.

Study	Selection				Comparability	Outcome		NOS scale
	**Representativeness of the sample**	**Sample size**	**Non-respondents**	**Ascertainment of exposure**		**Assessment of the outcome**	**Statistical test**	
Charakorn C *et al.* , 2011[23]	Selected group of users	Not justified	Comparability with non-respondents not established	Validated measurement tool*	Controls for the most important factor* and any additional factors*	Self –report*	Clearly described and association was measured appropriately*	5
Choi HCW *et al.*, 2013[11]	Somewhat representative*	Not justified	Comparability with non-respondents not established	Validated measurement tool*	Controls for the most important factor* and any additional factors*	Self –report*	Clearly described and association was measured appropriately*	6
Egawa-Takata T *et al.*, 2015[13]	Somewhat representative*	Not justified	Comparability with non-respondents not established	Non-validated measurement tool	Controls for the most important factor* and any additional factors*	Self –report*	Clearly described and association was measured appropriately*	5
Egawa-Takata T *et al.*, 2016[12]	Selected group of users	Not justified	Comparability with non-respondents not established	Non-validated measurement tool	Not investigated	Self –report*	Incomplete	1
Gu C *et al.*, 2015[8]	Somewhat representative*	Not justified	Comparability with non-respondents is established*	Validated measurement tool*	Controls for the most important factor* and any additional factors*	Self –report*	Clearly described and association was measured appropriately*	7
Hsu YY *et al.*, 2009[16]	Somewhat representative*	Not justified	Comparability with non-respondents is established*	Validated measurement tool*	Controls for the most important factor* and any additional factors*	Self –report*	Clearly described and association was measured appropriately*	7
Johnson DC *et al.*, 2014[22]	Somewhat representative*	Not justified	Comparability with non-respondents not established	Validated measurement tool*	Controls for the most important factor* and any additional factors*	Self –report*	Clearly described and association was measured appropriately*	6
Juntasopeepun P *et al.*,2012[24]	Somewhat representative*	Not justified	Comparability with non-respondents is established*	Validated measurement tool*	Controls for the most important factor* and	Self –report*	Clearly described and association was measured appropriately*	7

					any additional factors*			
Kang HS *et al.*, 2009[14]	Somewhat representative*	Justified*	Comparability with non-respondents not established	Validated measurement tool*	Not investigated	Self –report*	Clearly described and association was measured appropriately*	5
Kang HY *et al.*, 2011[15]	Somewhat representative*	Justified*	Comparability with non-respondents is established*	Validated measurement tool*	Not investigated	Self –report*	Clearly described and association was measured appropriately*	6
Kruiroongroj S *et al.*, 2014[25]	Somewhat representative*	Not justified	Comparability with non-respondents not established	Non-validated measurement tool	Not investigated	Self –report*	Clearly described and association was measured appropriately*	3
Li J *et al.*, 2009[9]	Somewhat representative*	Justified*	Comparability with non-respondents is established*	Non-validated measurement tool	Controls for the most important factor* and any additional factors*	Self –report*	Clearly described and association was measured appropriately*	7
Montgomery MP *et al.*, 2015[21]	Somewhat representative*	Not justified	Comparability with non-respondents not established	Non-validated measurement tool	Controls for the most important factor* and any additional factors*	Self –report*	Clearly described and association was measured appropriately*	5
Sam IC *et al.*, 2008[19]	Somewhat representative*	Not justified	Comparability with non-respondents not established	Non-validated measurement tool	Controls for the most important factor* and any additional factors*	Self –report*	Clearly described and association was measured appropriately*	5
Wong LP, 2010[20]	Somewhat representative*	Not justified	Comparability with non-respondents not established	Validated measurement tool*	Not investigated	Self –report*	Clearly described and association was measured appropriately*	4
Yen CF et al., 2011[17]	Selected group of users	Not justified	Comparability with non-respondents not established	Non-validated measurement tool	Controls for the most important factor* and any additional factors*	Self –report*	Clearly described and association was measured appropriately*	4
Young AM *et al.*, 2010[26]	Somewhat representative*	Not justified	Comparability with non-respondents not established	Non-validated measurement tool	Controls for any additional factor.*	Self –report*	Clearly described and association was measured appropriately*	4
Yu Y *et al.*, 2016[10]	Somewhat representative*	Not justified	Comparability with non-respondents not established	Non-validated measurement tool	Controls for the most important factor* and any additional factors*	Self –report*	Clearly described and association was measured appropriately*	5

Zhuang QY *et al*., 2016[18]	Somewhat representative*	Not justified	Comparability with non-respondents not established	Validated measurement tool*	Controls for the most important factor* and any additional factors*	Self –report*	Clearly described and association was measured appropriately*	6
